# Phytochemicals of *Periploca aphylla* Dcne. ameliorated streptozotocin-induced diabetes in rat

**DOI:** 10.1186/s12199-021-00962-0

**Published:** 2021-03-22

**Authors:** Umbreen Rashid, Muhammad Rashid Khan

**Affiliations:** Department of Biochemistry, Faculty of Biological Sciences, Quaid-i-Azam University, Islamabad, 45320 Pakistan

**Keywords:** Antidiabetic, Antioxidant, Anti-inflammatory, Insulin, Lipid profile

## Abstract

**Background:**

*Periploca aphylla* is used by local population and indigenous medicine practitioners as stomachic, tonic, antitumor, antiulcer, and for treatment of inflammatory disorders. The aim of this study was to evaluate antidiabetic effect of the extract of *P. aphylla* and to investigate antioxidant and hypolipidemic activity in streptozotocin (STZ)-induced diabetic rats.

**Methods:**

The present research was conducted to evaluate the antihyperglycemic potential of methanol extract of *P. aphylla* (PAM) and subfractions *n*-hexane (PAH), chloroform (PAC), ethyl acetate (PAE), *n*-butanol (PAB), and aqueous (PAA) in glucose-overloaded hyperglycemic Sprague-Dawley rats. Based on the efficacy, PAB (200 mg/kg and 400 mg/kg) was tested for its antidiabetic activity in STZ-induced diabetic rats. Diabetes was induced via intraperitoneal injection of STZ (55 mg/kg) in rat. Blood glucose values were taken weekly. HPLC-DAD analysis of PAB was carried out for the presence of various polyphenols.

**Results:**

HPLC-DAD analysis of PAB recorded the presence of rutin, catechin, caffeic acid, and myricetin. Oral administration of PAB at doses of 200 and 400 mg/kg for 21 days significantly restored (*P* < 0.01) body weight (%) and relative liver and relative kidney weight of diabetic rats. Diabetic control rats showed significant elevation (*P* < 0.01) of AST, ALT, ALP, LDH, total cholesterol, triglycerides, LDL, creatinine, total bilirubin, and BUN while reduced (*P* < 0.01) level of glucose, total protein, albumin, insulin, and HDL in serum. Count of blood cells and hematological parameters were altered in diabetic rats. Further, glutathione peroxidase, catalase, superoxide dismutase, glutathione reductase, and total soluble protein concentration decreased while concentration of thiobarbituric acid reactive substances and percent DNA damages increased (*P* < 0.01) in liver and renal tissues of diabetic rats. Histopathological damage scores increased in liver and kidney tissues of diabetic rats. Intake of PAB (400 mg/kg) resulted in significant improvement (*P* < 0.01) of above parameters, and results were comparable to that of standard drug glibenclamide.

**Conclusion:**

The result suggests the antihyperglycemic, antioxidant, and anti-inflammatory activities of PAB treatment in STZ-compelled diabetic rat. PAB might be used as new therapeutic agent in diabetic patients to manage diabetes and decrease the complications.

## Background

Diabetes mellitus (DM) is a fast-growing disease in the world with serious health issues and complications. There are different factors which can contribute towards the onset of diabetes type 2 in population. Among these factors, aging, physical inactivity, overweight, heredity disposition, over 40 years of age, lifestyle, and poor eating habits have been implicated in its increasing prevalence [1]. Its prevalence is increasing quite rapidly in the world, and it has been projected to be 69% from 2010 to 2030 in adults of developing countries [2]. Pakistan also belongs to developing countries having sharp rise in diabetic type 2 patients. Meta-analysis of studies revealed high prevalence of diabetic (14.62%) and pre-diabetic individuals (11.43%) in Pakistan [3]. DM has become a challenge for human health and causes a significant loss to economic health as well [4].

Diabetes type 2 is usually associated with hyperglycemic conditions. During early stages of DM, inadequate concentration of insulin and/or inefficient response of the body to insulin metabolism of lipids, proteins, and carbohydrates are disturbed. And with advancement of the disease, insulin level fails to sustain requirement of the body. This leads to impairment of the immune system and consequently results in insulitis and pancreatitis. Further, inefficacy of the inflammatory response may cause diabetes [5]. Oxidative stress, excessive production of reactive oxygen species (ROS), is one of several factors involved in pathogenesis of diabetes leading to pancreatic β-cell injuries due to weak intrinsic free radical scavenging potential [6, 7]. Patients with DM experience different pathological complications of the cardiovascular, eyes, brain, and kidneys in both type 1 and type 2 diabetes. These impediments predisposed DM patients to morbidity and mortality [8]. Therefore, appropriate interventions could help in preventing or delaying the onset of diabetes.

In traditional health care systems, plants or their extracts have long been in practice for various disorders including diabetes. The genus *Periploca* belongs to family Apocynaceae with ten species. Species of *Periploca* are utilized worldwide by local communities for diverse disorders for their cardiotonic, anti-inflammatory, immunosuppressive, antitumor, antimicrobial, antioxidant, and other properties [9]. In Pakistan, this genus is presented by three species: *P. aphylla*, *P. hydaspidis*, and *P. forrestii*. Local populations in different areas of Pakistan use *P. aphylla* for various disorders including swollen joints, cough, flu, constipation, ulcer, skin diseases [10], spleen enlargement, and in rheumatism, and as stomachic, tonic, antidiarrheal, diuretic, and laxative [11–16]. *Periploca laevigata* treatment reduced the diabetes-related parameters in rat [17]. Extract of *P. hydaspidis* showed antioxidant and hepatoprotective activities against carbon tetrachloride-induced oxidative stress in rat [18] while ethanol extract of *P. angustifolia* showed hypoglycemic activity in diabetic rats [19].

Different alternative therapies having antihyperglycemic, antilipidemic, and antioxidant activities should be targeted for clinic settings to prevent or delay the sufferings and complications of diabetes type 2 patients. In developing countries, most of the population depends upon plant-based remedies for different ailments. The traditional medicines also gained popularity worldwide because of fewer side effects. Different phytochemical classes especially polyphenols in many plants has gained much attention as alternative therapeutics in many disorders. Role of such plants in the management of diabetes and amelioration of complications has been explained in different studies [7, 20]. The antioxidant and anti-inflammatory effects of *Periploca* genus have been evaluated in different studies [12, 18, 19]. In this investigation, we have analyzed the polyphenolic constituents and evaluated PAB for hypoglycemic and antioxidant activity in streptozotocin-induced diabetic rats.

## Materials and method

### Plant collection

The aerial parts of *P. aphylla* were collected from Margalla Hills Islamabad. The specimen was deposited (058608) at the Pakistan Museum of Natural History, Islamabad, Pakistan, after authentication by Dr. Saleem Ahmad, Curator, Pakistan Museum of Natural History, Islamabad, Pakistan.

### Preparation of extract

Extract of *P. aphylla* (1.5 kg) was obtained by maceration of powder in 3 L of methanol for 7 days. Methanol extract (PAM) was obtained after drying the filtrate at 40 °C in a rotary vacuum evaporator. A portion of PAM was sub-fractionated into *n*-hexane (PAH), chloroform (PAC), ethyl acetate (PAE), *n*-butanol (PAB), and soluble aqueous portion (PAA) dried as above and stored at 4 °C until use.

#### HPLC-DAD analysis of PAB

In PAB, separation and estimation of polyphenols were carried out on HPLC system equipped with Agilent 1200 diode array detector. The PAB was resolved by using methanol-acetonitrile-water-acetic acid (10:5:85:1) as mobile phase A while B constituted of methanol-acetonitrile-acetic acid (60:40:1). The column used was Discovery-C18 analytical column (250 × 4.6 mm, 5-μm particle size, Supelco, USA). The phases were adjusted as of 1.0 ml/min flow with gradient of time 0–20 min for 0 to 50% B, 20–25 min for 50 to 100% B, and then isocratic 100% B till 30 min. The standard compounds and PAB were injected (20 μl) separately. The resolution of rutin and gallic acid was studied at 257 nm, catechin at 279 nm, caffeic acid at 325 nm, and quercetin, myricetin, and kaempferol at 368 nm. The content of polyphenols was estimated from peak areas of HPLC chromatograms on the basis of three replicates.

### Experimental animals

At the Primate Facility of Quaid-i-Azam University, Islamabad, Pakistan, the Sprague-Dawley male rats (150–200 g) were acclimatized at 12-h light and 12-h dark cycle at 25±30 °C, and standard diet along with water ad libitum was provided to rats. The experiment was performed after authentication of the ethical committee of the institute (Bch#245) for use of rats in experimental purposes.

### Glucose tolerance studies in normal rats

From overnight-fasted (15 h) rats, eight groups (6 in each) were made. DMSO 5% (1 ml/kg) was administered to control rats while rats of other groups received 200 mg/kg of PAM, PAH, PAC, PAE, PAB, and PAA and 10 mg/kg of glibenclamide to each rat and after 30 min by 10 g/kg of 5% dextrose. Concentration of glucose (mg/dl) in the blood was monitored by tail puncturing after various intervals: 0, 1, 2, and 4 h of treatment.

### Glucose tolerance studies in diabetic rats

In overnight-fasted rats (15 h), diabetes was induced by intraperitoneal injection of 210 mg/kg of nicotinamide and after 15 min with 55 mg/kg of streptozotocin in 0.9% saline [21]. Control rats were injected with 0.9% of saline. Rats having blood glucose level > 230 mg/dl after 48 h of this treatment were used to evaluate antihyperglycemic activity of extract/fractions as above.

### Multiple-dose studies

For induction of diabetes in Sprague-Dawley male rats, above model was followed, and antidiabetic activity of PAB treatment was evaluated in chronic multiple-dose experiment. The following treatments were administered for 21 days to each rat. The extract and standard drug was dissolved in DMSO for administration to rats.

Group 1: normal rats treated with 1 ml/kg of vehicle (5% DMSO)

Group 2: diabetic rats treated with only 1 ml/kg of vehicle (5% DMSO)

Group 3: diabetic rats treated with PAB (200 mg/kg equal to 2.39 mg rutin)

Group 4: diabetic rats treated with PAB (400 mg/kg equal to 4.78 mg rutin)

Group 5: diabetic rats treated with glibenclamide (10 mg/kg)

The glucose estimation was done after 1, 7, 14, and 21 days (as above). Body weight of rat was recorded at 1 and after 21 days in fasting condition, and percent change in body weight was determined for each rat.

### Assessment of blood parameters and other metabolites

Animals were anesthetized with 1% chloralose and 25% urethane at a dose of 1 mg/kg, and blood was collected after sacrifice from cardiac puncture in BD vacutainer plus serum tubes. Different parameters of hemoglobin, count of blood cells, were estimated in the 1st portion of blood by CELL-DYN Ruby Automated 5 part Hematology Analyzer (Abbott Diagnostics, Germany). In serum obtained from 2nd portion of blood liver function enzymes, lipid profile and kidney function markers were assessed with AMP diagnostic kits (Stattogger Strasse 31b 8045 Graz, Austria).

### Assessment of pro-inflammatory cytokines in serum

By using the manufacturer protocol, we have estimated the concentration of various cytokines: tumor necrosis factor-alpha (TNF-α) RayBio-Tech, Inc. (Norcross, GA, USA), IL-6 (interleukin-6), and TGF-β1 (transforming growth factor-beta 1) of Aviscera-Bioscience, Inc. (Santa Clara, CA, USA) in serum.

### Antioxidant profile in hepatic and renal tissues

Supernatant obtained after centrifugation from homogenate (10%; v/v) of liver and kidney tissues was used for different assays. Lowry et al. [22] protocol was used to assess the concentration of protein in liver and kidney tissues. Different assays were applied for the estimation of catalase activity [23], superoxide dismutase activity [24], glutathione reductase activity [25], and for glutathione peroxidase activity [26], reduced glutathione [27] while lipid peroxidation (TBARS) with method of Iqbal et al. [28]. DNA damages were also determined in various treated groups in tissues of the liver and kidneys.

### Histology of the liver and kidneys

Paraffin wax-embedded tissues of the liver and kidneys were sectioned at 4 μm and stained with hematoxylin/eosin. The sections were studied under light microscope (DIALUX 20 EB) at ×40 magnifications. Different histological injuries observed in the liver and renal tissues were recorded in 0–3 score (0, no injury; 1, mild injury; 2, medium injury; 3, severe injury).

### Statistical analysis

The data of the experiment was presented in terms of mean and standard error of the mean. To estimate the variability among treatments, one way ANOVA was applied by computer software GraphPad prism 4.0. Significance among groups was determined by Tukey’s HSD test at *P*-value ≤ 0.05.

## Results

### HPLC-DAD studies

**Fig. 1:**
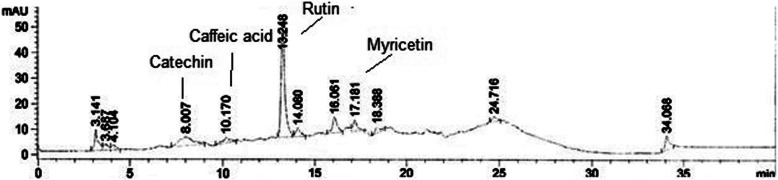
HPLC analysis of *n*-butanol fraction of *P. aphylla*

The HPLC-DAD analysis of PAB showed the presence of rutin, catechin, caffeic acid, and myricetin at retention times (minutes) of 13.248, 8.007, 10.170, and 17.181 at wavelengths of 257, 279, 325, and 368 nm, respectively. The maximum amount of rutin (11.950 ± 0.02 μg/mg) was recorded and followed by catechin (0.641 ± 0.001 μg/mg), myricetin (0.441 ± 0.005 μg/mg) and caffeic acid (0.278 ± 0.001 μg/mg) (Fig. 1).

### Hypoglycemic effect in normal rats

**Fig. 2:**
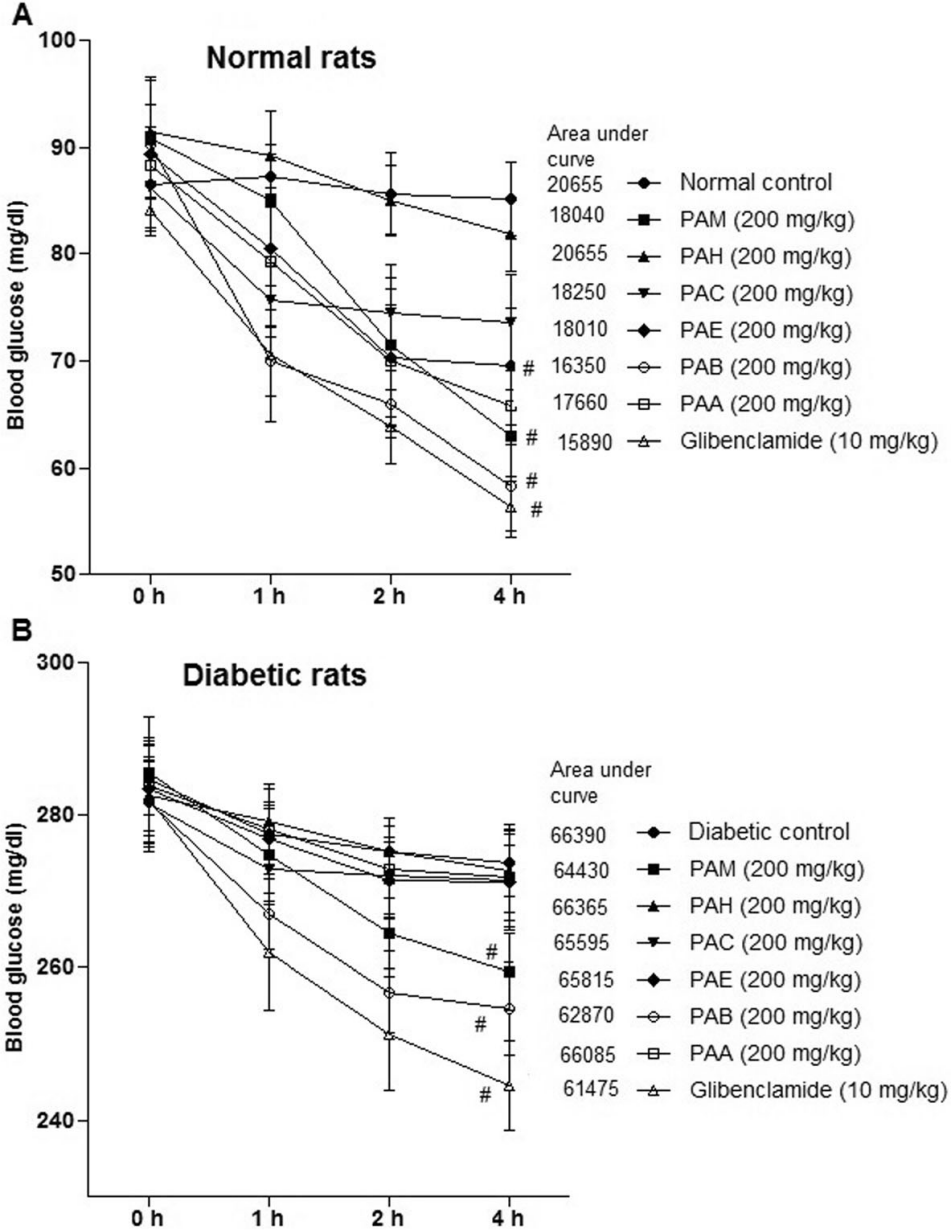
Effect of PAM and fractions on glucose tolerance in rat. **a** Normal rats. **b** Diabetic rats. Data represent mean±SEM (6 number). PAM, *P. aphylla* methanol extract; PAH, *n*-hexane fraction of PAM; PAC, chloroform fraction of PAM; PAE, ethyl acetate fraction of PAM; PAB, *n*-butanol fraction of PAM; PAA, residual soluble aqueous fraction of PAM; glibenclamide (standard drug). Number sign  indicates significance from control group at *P* < 0.01 level

Figure 2 a shows the antihyperglycemic effects of PAM and subfractions, PAH, PAC, PAE, PAB and PAA (200 mg/kg), in glucose-loaded hyperglycemic rats. According to the results, at 0 h, there were non-significant differences (*P* = 0.0624), while at 1 h (*P*= 0.0462), 2 h (*P* = 0.0087), and at 4 h (0.0028) significant differences among treatment were determined for glucose concentration in serum of rat. PAH, PAC, and PAE exhibited the antihyperglycemic (*P* < 0.01) effect at 2 h and 4 h after the glucose treatment compared to control. PAM, PAA, and PAB were found to produce hypoglycemia at 1 h, 2 h, and 4 h (*P* < 0.01) of treatment. As shown in Fig. 2a after administration of PAB, lower values for area under a curve (16,350 units) were obtained as compared with extract and subfractions: PAM (18,040 units), PAH (20,655 units), PAC (18,250 units), PAE (18,010 units), and PAA (17,660 units).

### Hypoglycemic effect in diabetic rats

Hypoglycemic effects of extract/fractions in diabetic rats are shown in Fig. 2b. Blood glucose decreased (*P* < 0.05) with PAM and subfractions at 1 h as compared with diabetic control. After 2 h, values of blood glucose have not been altered (*P* > 0.05) with PAH, PAC, PAE, and PAA as compared with diabetic control rats. PAB and PAM decreased (*P* < 0.01) the values of blood glucose at 2 h and 4 h. The area under a curve for diabetic control rats, PAM treated, and PAB administered was 66,390 units, 64,430 units, and 62,870 units, respectively. Comparatively, the activity of PAB was better than that of the activity exhibited by other extract/subfractions (Fig. 2b).

### Effect of PAB in multiple-dose study

**Fig. 3:**
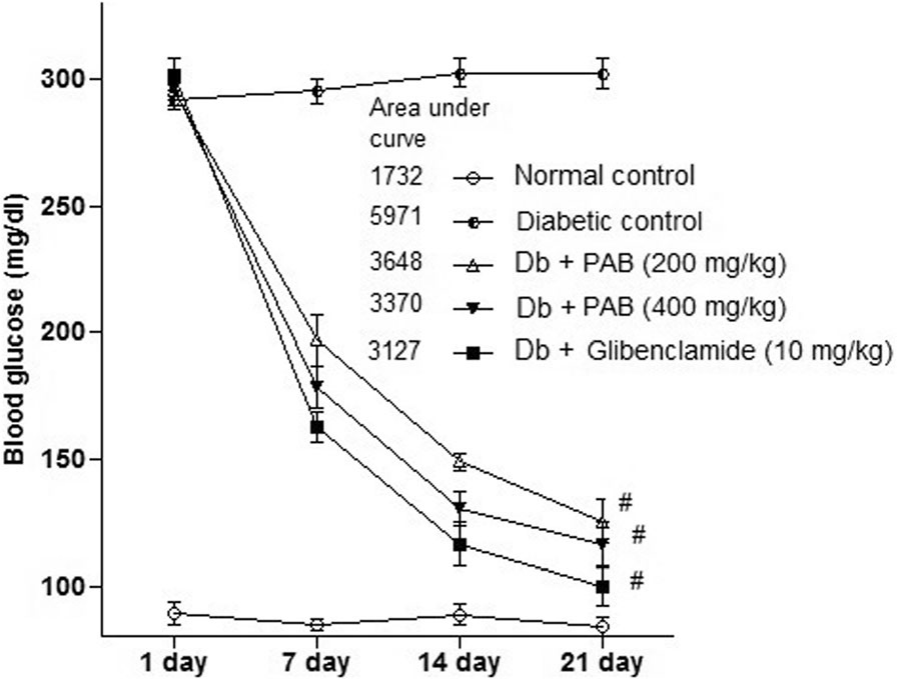
Effect of PAB in a 21-day experiment on diabetic rats. Data represent mean±SEM (6 number). PAB, *n*-butanol fraction of PAM; number sign indicates significance from diabetic control group at *P* < 0.01 level

To evaluate the hypoglycemic activity of PAB in diabetic rat, concentration of glucose in serum was recorded at 1 day, 7 days, 14 days, and 21 days (Fig. 3). The result indicated significant differences among treatments on glucose concentration in serum of rat after 7 days (*P* = 0.0081), 14 days (*P* = 0.0062), and 21 days (*P* = 0.0014) of treatment. STZ-compelled diabetic rats exhibited area under a curve of 5971 units as compared to area under a curve of 1732 units of normal control rats (Fig. 3). The area under a curve for PAB (200 mg/kg) determined was 3648 units while at 400 mg/kg of PAB it was 3370 units as compared to area under a curve of 3127 units with standard drug glibenclamide.

### Effect of PAB on body weight and relative weight of the liver and kidney

The putative protective effects of PAB on general toxicity were evaluated at 21 days in diabetic rats. The results indicated significant differences among treatments for increase in body weight (*P* = 0.0084), relative liver weight (*P* = 0.0027), and relative kidney weight (*P* = 0.0039) of rat after 21 days of treatment (Table 1). The body weight, relative liver weight, and relative kidney weight were lower (*P* < 0.01) in diabetic rats than in normal control rats. Treatment with PAB obviously (*P* < 0.01) increased the body weight, relative liver weight, and relative kidney weight of diabetic rats.

**Table 1:** Effect of butanol extract of *P. aphylla* on percent increase in body weight in control and diabetic rats

Treatment (mg/kg)	Percent increase in body weight	Absolute liver weight (g)	Relative liver weight (% to body weight)	Absolute kidney weight (g)	Relative kidney weight (% to body weight)
Normal control	34.70 ± 1.82	5.28 ± 0.94	0.052 ± 0.001	1.56 ± 0.04	0.015 ± 0.0001
Diabetic control	20.76 ± 1.21^*^	3.42 ± 0.68	0.034 ± 0.001^*^	0.42 ± 0.01	0.004 ± 0.0001^*^
Db + PAB 200	25.29 ± 0.56^*#^	4.12 ± 0.54	0.041 ± 0.001^*#^	0.83 ± 0.08	0.008 ± 0.0001^*#^
Db + PAB 400	28.98 ± 1.26^*#^	4.88 ± 0.37	0.048 ± 0.002^*#^	1.32 ± 0.02	0.013 ± 0.0003^#^
Db + Glib 10	31.06 ± 0.98^*#^	5.13 ± 0.75	0.051 ± 0.002^#^	1.51 ± 0.02	0.015 ± 0.0002^#^

Values are expressed as mean ± SEM (06). “^*^” indicates significance difference from normal control whereas “^#^” from diabetic control at *P* < 0.01 level

*Db* diabetic, *PAB P. aphylla n*-butanol extract, *Glib* glibenclamide

### Effect of PAB on hematological parameters

The results indicated significant differences among treatments for RBC (*P* = 0.0012), Hb (*P* = 0.0025), MCV (*P* = 0.0032), MCHC (*P* = 0.0074), and MCH (*P* = 0.014) values. In diabetic rat, RBC, Hb, MCV, MCHC, and MCH decreased significantly (*P* < 0.01) as compared with normal control rats. After treatment with PAB at different concentrations (200 mg/kg and 400 mg/kg) or glibenclamide to diabetic rats, RBC, Hb, MCV, MCHC, and MCH were increased (*P* < 0.01) compared with that in the diabetic control rats (Table 2).

**Table 2:** Effect of *P. aphylla n*-butanol extract on full blood counts in control and diabetic rats

Treatment (mg/kg)	RBC (×10^**12**^/l)	Hb (g/l)	MCV (fl)	MCHC (g/dl)	MCH (pg)
Normal control	8.76 ± 0.52	15.56 ± 0.84	58.33 ± 0.15	32.11 ± 0.46	18.61 ± 2.67
Diabetic control	6.27 ± 0.45^*^	10.10 ± 0.59^*^	52.85 ± 1.22^*^	20.45 ± 0.52^*^	15.75 ± 2.41^*^
Db + PAB 200	7.78 ± 0.56^*#^	12.67 ± 0.84^*#^	54.38 ± 2.56	26.56 ± 1.33^*#^	17.65 ± 0.12
Db + PAB 400	8.61 ± 1.65	14.72 ± 1.78^#^	57.42 ± 1.25^#^	29.73 ± 1.45^#^	19.44 ± 1.77^#^
Db + Glib 10	8.25 ± 1.29^#^	14.21 ± 0.73^#^	57.34 ± 1.43^#^	30.14 ± 0.55^#^	19.58 ± 2.86^#^

Values are expressed as mean ± SEM (06). “^*^” indicates significant difference from normal control whereas “^#^” from diabetic control at *P* < 0.01 level

*Db* diabetic, *PAB P. aphylla n*-butanol extract, *Glib* glibenclamide

### Effect of PAB on count of blood cells

The count of different blood cells was recorded to determine whether PAB administration restores the count of these cells in diabetic rats. The analysis of blood cell count showed significant differences among treatments for WBC (*P* = 0.0054), platelets (*P* = 0.0061), lymphocytes (*P* = 0.0028), and neutrophils (*P* = 0.0018) in the blood of rat. In diabetic control rats, count of WBC, platelets, lymphocytes, and neutrophils was significantly (*P* < 0.01) decreased as compared with normal control rats (Table 3). After treatment with PAB (200 mg/kg and 400 mg/kg) or glibenclamide to diabetic rats, count of WBC, platelets, lymphocytes, and neutrophils in the blood significantly (*P* < 0.01) increased as compared with diabetic control rats (Table 3).

**Table 3:** Effect of *P. aphylla* butanol extract on full blood counts in control and diabetic rats

Treatment (mg/kg)	WBC (10^**3**^/μl)	Platelets (× 10^**3**^/μl)	Lymphocytes (%)	Neutrophils (%)
Normal control	7.85 ± 0.43	872.15 ± 16.83	69.47 ± 2.12	28.41 ± 1.64
Diabetic control	2.17 ± 0.89^*^	84.67 ± 2.78^*^	10.38 ± 1.75^*^	8.73 ± 1.85^*^
Db + PAB 200	3.69 ± 0.47^*^	132.54 ± 21.61^*#^	50.81 ± 1.67^*#^	22.58 ± 1.39^*#^
Db + PAB 400	6.91 ± 0.24^#^	257.49 ± 13.85^*#^	66.92 ± 2.18^#^	29.42 ± 0.62^#^
Db + Glib 10	6.23 ± 0.16^#^	187.35 ± 18.44^*#^	64.52 ± 1.36^*#^	26.52 ± 0.78^*#^

Values are expressed as mean ± SEM (06). “^*^” indicates significant difference from normal control whereas “^#^” from diabetic control at *P* < 0.01 level

*Db* diabetic, *PAB P. aphylla n*-butanol extract, *Glib* glibenclamide

### Effect of PAB on lipid profile in serum

**Fig. 4:**
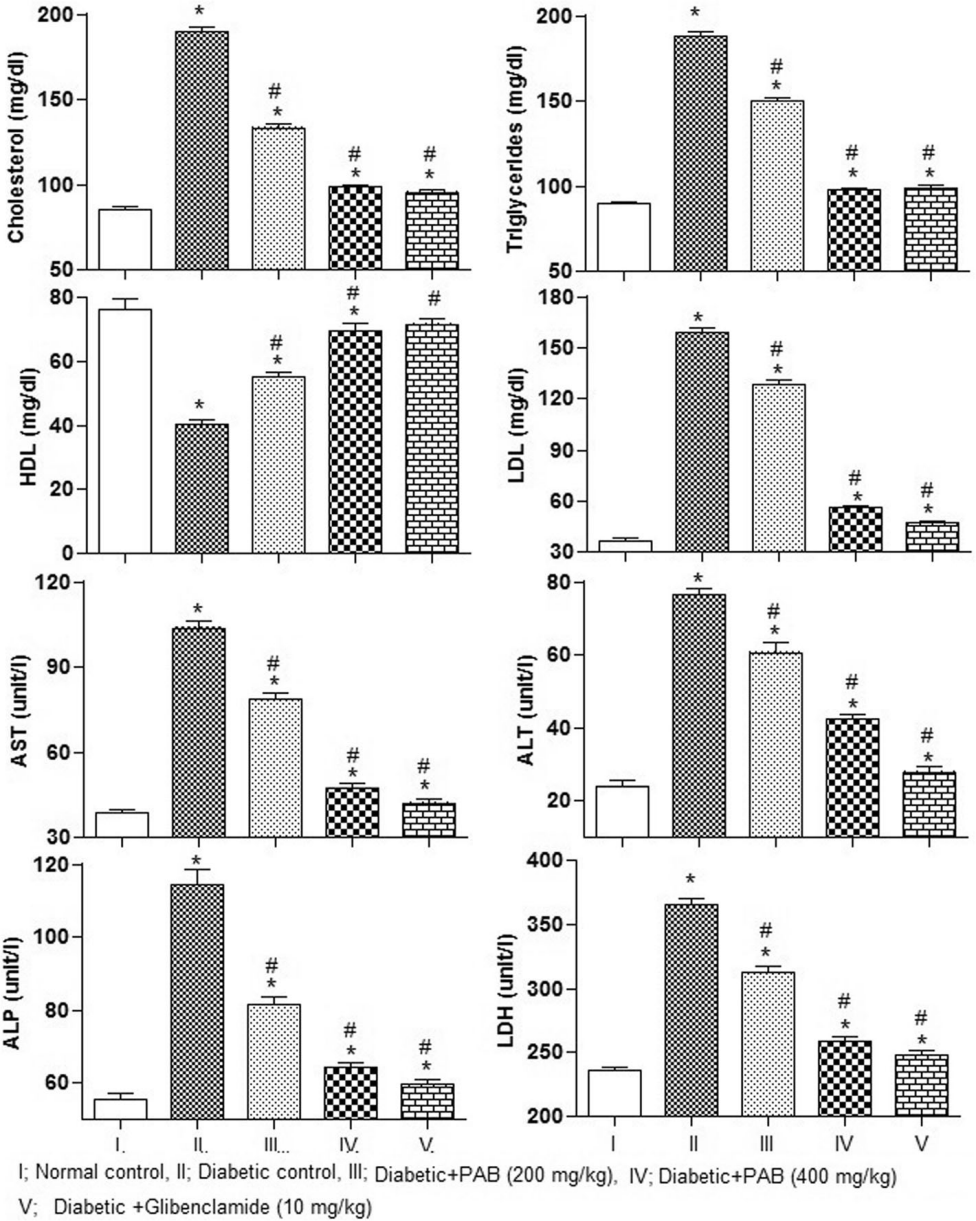
Effect of PAB in a 21-day experiment on lipid profile and hepatic markers in diabetic rats. Data represent mean±SEM (6 number). PAB, *n*-butanol fraction of PAM; asterisk indicates significance from control group at *P* < 0.01 level; number sign indicates significance from diabetic control group at *P* < 0.01 level

The effect of different treatments on lipid profile of diabetic rats is shown in Fig. 4. The results showed significant differences among treatments for level of triglycerides (*P* = 0.0071), cholesterol (*P* = 0.0024), LDL (*P* = 0.0049), and HDL (*P* = 0.0067) in serum of rat after 21 days of treatment. In STZ-compelled diabetic rats, significantly (*P* < 0.01) elevated level of triglycerides, cholesterol, and LDL in serum as compared to normal control rats was recorded (Fig. 4). In contrast, level of HDL (*P* < 0.01) decreased in diabetic control rats as compared with normal control rats. The extract PAB (200 mg/kg and 400 mg/kg) significantly reduced triglycerides, cholesterol, and LDL while LDL increased in diabetic rats (*P* < 0.01) compared with the diabetic control rats.

### Effect of PAB on liver function markers

The results showed significant differences among treatment for AST (*P* = 0.0057), ALT (*P* = 0.0038), ALP (*P* = 0.0047), and LDH (*P* = 0.0034) in serum of rat. The average level of liver markers, AST, ALT, ALP, and LDH, of diabetic control rats was higher (*P* < 0.01) than in normal control rats (Fig. 4). PAB (200 mg/kg and 400 mg/kg) had significantly decreased (*P* < 0.01) AST, ALT, ALP, and LDH, compared with diabetic control rats.

### Effect of PAB on kidney function markers and insulin

**Fig. 5:**
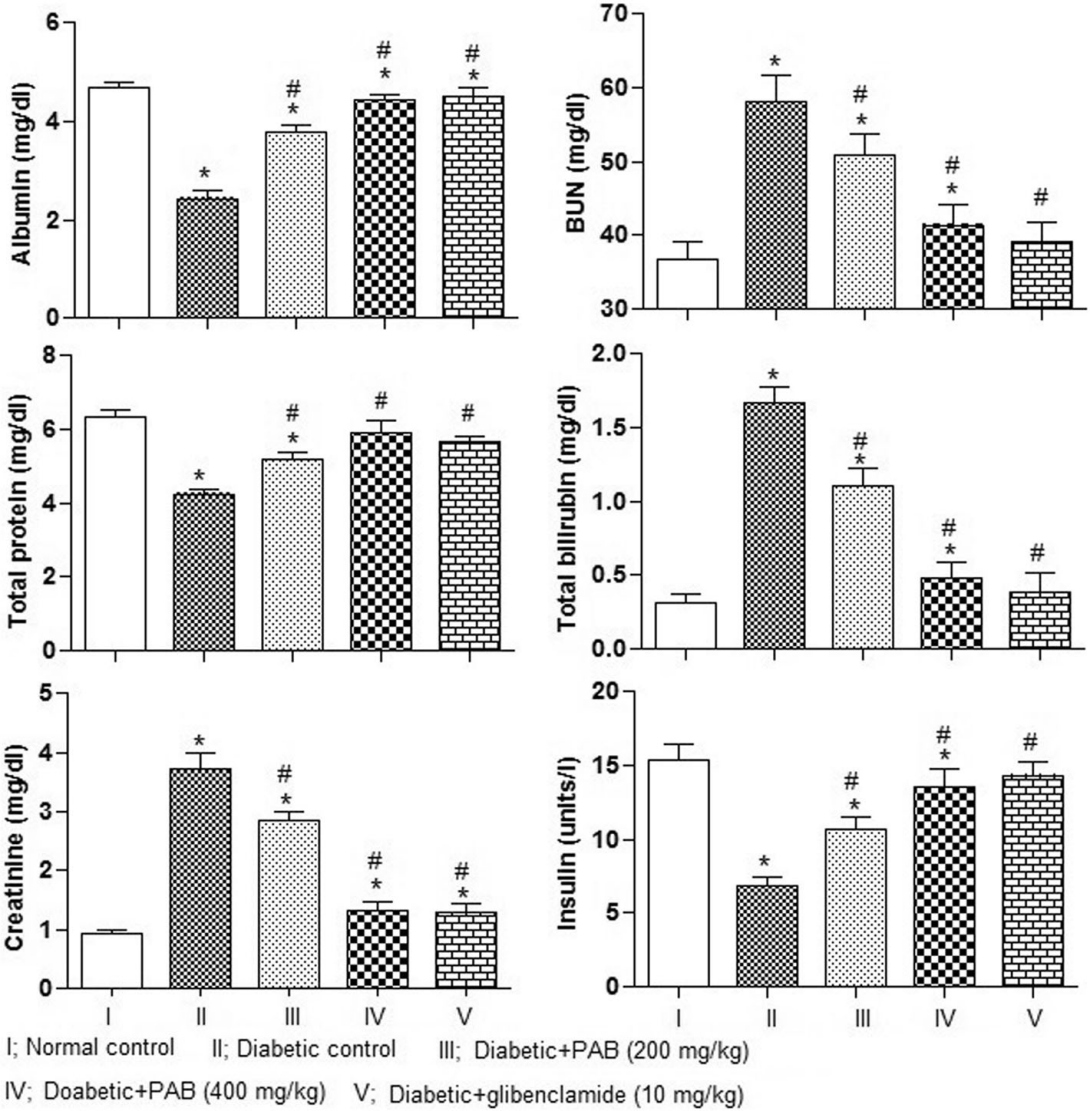
Effect of PAB in a 21-day experiment on kidney function markers and insulin in diabetic rats. Data represent mean±SEM (6 number). PAB, *n*-butanol fraction of PAM; asterisk indicates significance from control group at *P* < 0.01 level, number sign indicates significance from diabetic control group at *P* < 0.01 level

Significant differences among various treatments were determined for blood urea nitrogen (*P* = 0.0135), creatinine (*P* = 0.0076), total bilirubin (*P* = 0.0043), total proteins (*P* = 0.0063), albumin (*P* = 0.0047), and insulin (*P* = 0.0082) in rat. In diabetic control rats, there are significantly (*P* < 0.01) higher average values of blood urea nitrogen, creatinine, and total bilirubin while significantly (*P* < 0.01) lower average values of total proteins, albumin, and insulin as compared to normal control rats were recorded (Fig. 5). The PAB treatment (200 mg/kg and 400 mg/kg) to diabetic rat decreased (*P* < 0.01) blood urea nitrogen, creatinine, and total bilirubin, compared with diabetic control rats. On the contrary, treatment of PAB significantly (*P* < 0.01) increased the concentration of total proteins, albumin, and insulin, compared with diabetic control rats.

### Effect of PAB on antioxidants in the liver and kidney

**Fig. 6:**
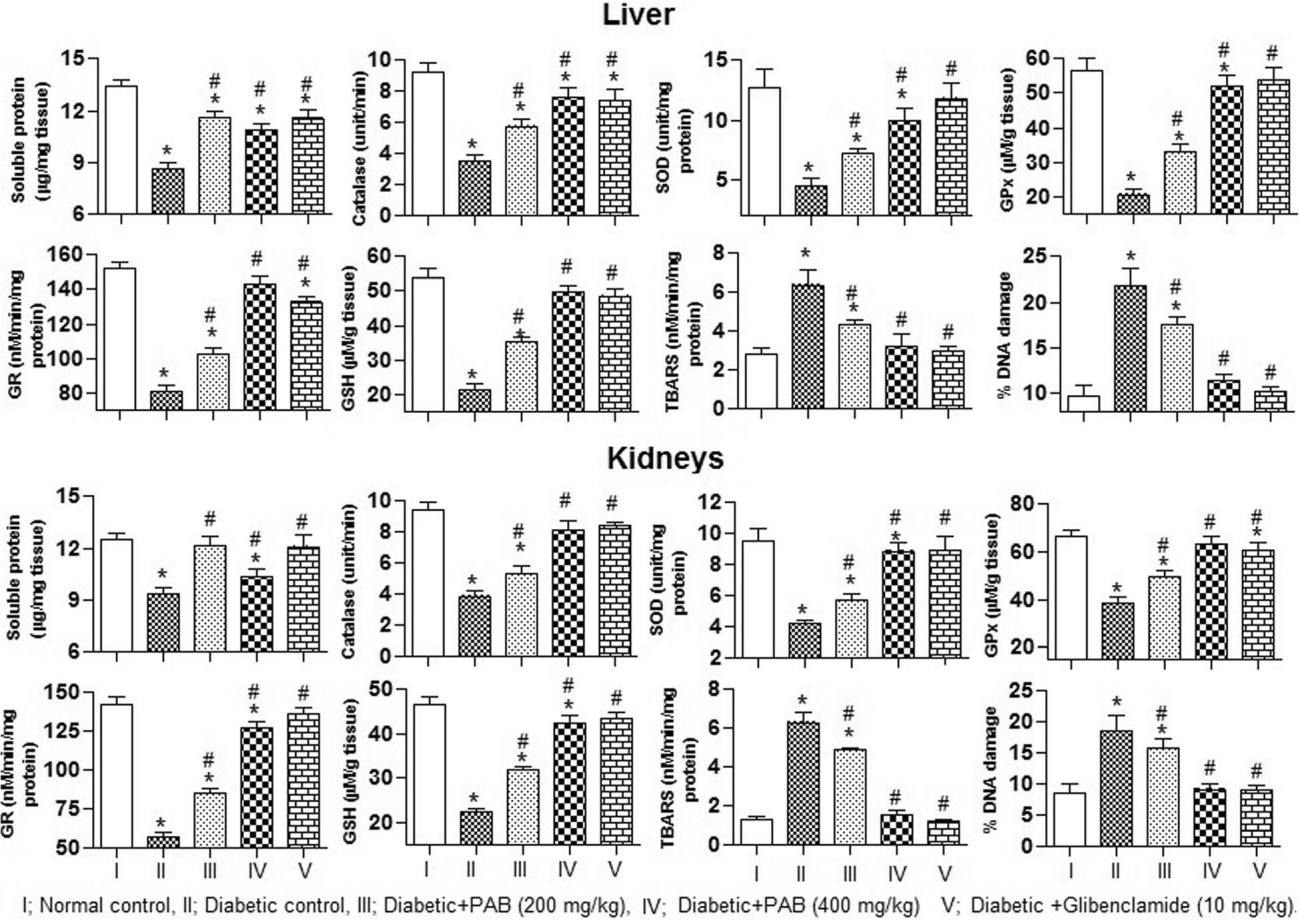
Effect of PAB in a 21-day experiment on antioxidant profile, lipid peroxidation, and DNA damages in diabetic rats. Data represent mean±SEM (6 number). PAB, *n*-butanol fraction of PAM; asterisk indicates significance from control group at *P* < 0.01 level; number sign indicates significance from diabetic control group at *P* < 0.01 level

To evaluate the antioxidant potential of PAB in diabetic rats, we measured level of activity of antioxidant markers in the liver and kidney samples of rat. And significant differences among treatments for the activity level of CAT (*P* = 0.0081), SOD (*P* = 0.0025), POD (*P* = 0.0037), GPx (*P* = 0.0046), GR (*P* = 0.0057), and concentration of soluble protein (*P* = 0.0033) in hepatic tissues of rat were determined in this study. Treatment with STZ alone induced a significant (*P* < 0.01) decrease in the activity level of CAT, SOD, POD, GPx, GR, and also concentration of soluble protein in hepatic tissues of rat as compared to the normal control rats (Fig. 6). Also consistent with above results, significant differences among treatment for activity level of CAT (*P* = 0.0024), SOD (*P* = 0.0039), POD (*P* = 0.0084), GPx (*P* = 0.0062), GR (*P* = 0.0073), and concentrations of soluble protein (*P* = 0.0092) were recorded in renal tissues of rat. However, decrease in activity of enzymatic antioxidants and accumulation of soluble protein was restored (*P* < 0.01) with PAB and glibenclamide administration to STZ-induced diabetic rats (Fig. 6).

### Effect of PAB on lipid peroxidation and DNA damages

To evaluate the extent of damages with STZ and restoration potential of PAB, we measured level of GSH, TBARS, and % DNA damages in the liver and kidney samples of rat. The analysis of data indicated significant differences among treatments for concentration of GSH (*P* = 0.0083), TBARS (*P* = 0.0044), and % DNA damages (*P* = 0.0068) in liver tissues of rat. Rats treated with STZ alone (diabetic control rats) demonstrated a significant (*P* < 0.01) decrease in the concentration of GSH, while an increase in the level of TBARS and % DNA damages in liver samples upon normal control rats (Fig. 6). This study also recorded significant differences among treatments for the level of GSH (*P* = 0.0073), TBARS (*P* = 0.0087), and % DNA damages (*P* = 0.0043) in renal tissues of rat. In STZ-induced diabetic rats, the level of GSH decreased whereas concentration of TBARS and % DNA damages increased (*P* < 0.01) in renal tissues as compared to normal control rats. The altered level of above markers was restored towards the normal rats with administration of glibenclamide (10 mg/kg) and PAB (200 mg/kg and 400 mg/kg) to diabetic rats (Fig. 6).

### Effect of PAB on liver histology

**Fig. 7:**
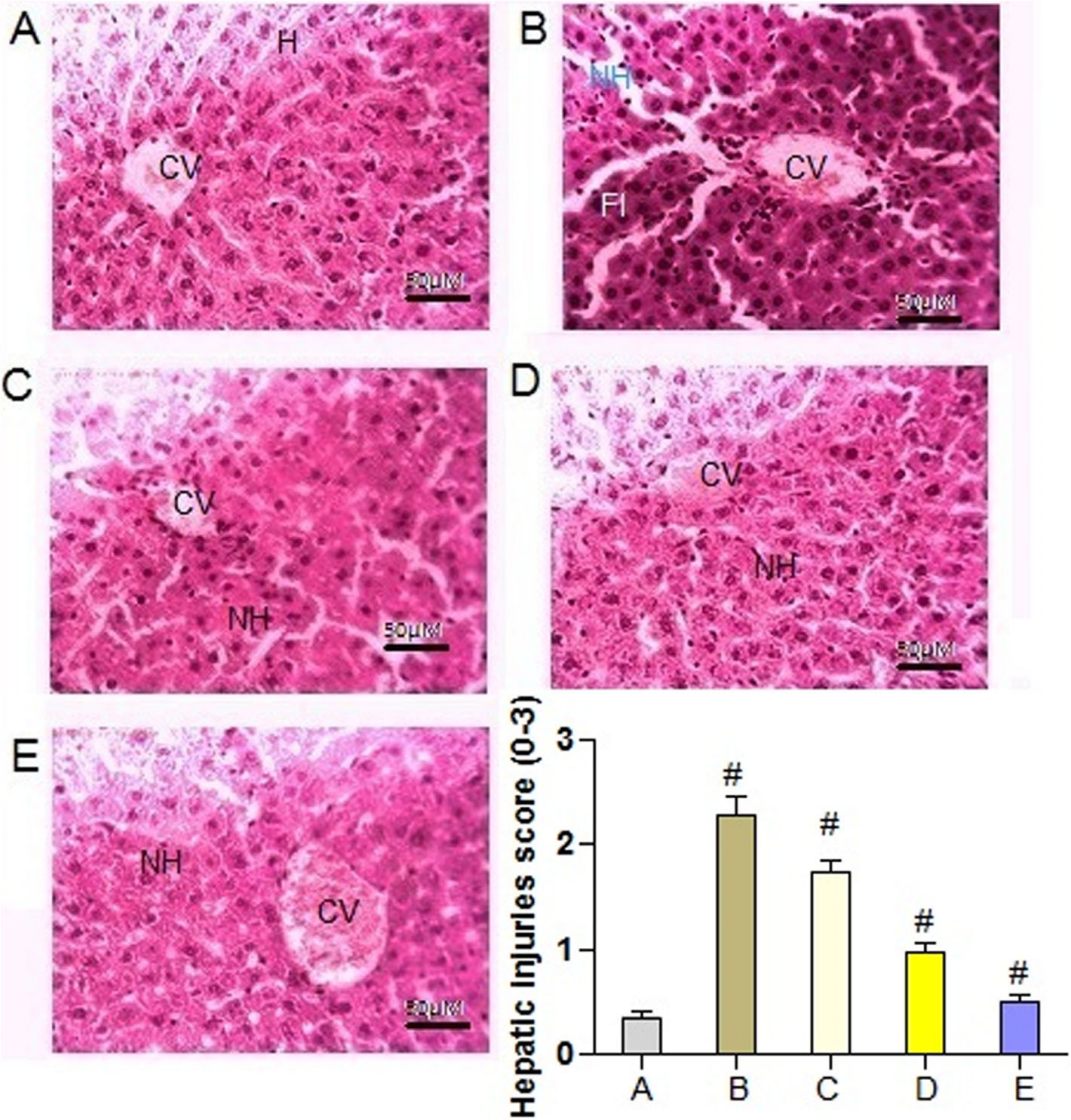
×40. Effect of PAB in a 21-day experiment on liver histopathology in diabetic rats. **a** Normal control. **b** Diabetic control. **c** Diabetic + PAB (200 mg/kg). **d** Diabetic + PAB (400 mg/kg), diabetic + glibenclamide (10 mg/kg). CV, central vein; HI, fatty infiltration; NH, necrotic hepatocytes; H, Normal hepatocytes. Number sign indicates significance from control group at *P* < 0.01 level

The histology of liver tissues in normal control rats exhibited normal sinusoidal cards of hepatocytes, portal tracts with triad, and central vein. These were accompanied with hepatic artery, portal vein, and bile duct (Fig. 7a). However, the histopathology of diabetic rats exhibited distorted cellular arrangement around the central vein, necrotic hepatocytes, and with fatty infiltration (Fig. 7b). The level of necrosis was reduced, and the cellular arrangement around the central vein was brought back to normal by the treatment of *P. aphylla* butanol extract (200 and 400 mg/kg) (Fig. 7c and d). Administration of glibenclamide (10 mg/kg) also ameliorated the changes induced in the liver of streptozotocin-treated rats (Fig. 7e). Histopathological alterations were also recorded in score of 0–3. In diabetic rats, significantly (*P* = 0.0067) higher score of injuries were noticed as compared to normal control rat. However, scores of these injuries were decreased in PAB- and glibenclamide (10 mg/kg)-treated diabetic rats.

### Effect of PAB on renal histopathology

**Fig. 8:**
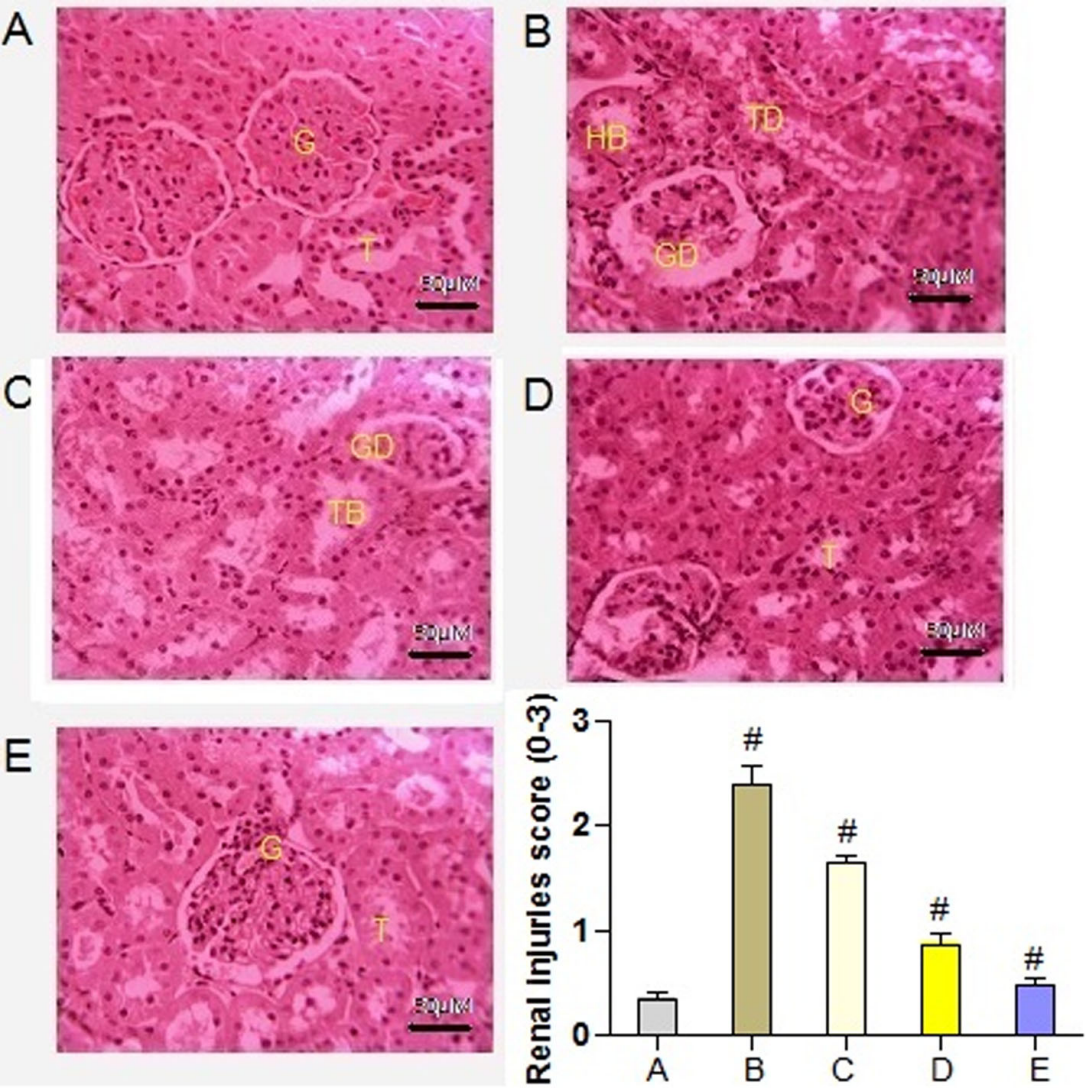
×40. Effect of PAB in a 21-day experiment on kidney histopathology in diabetic rats. **a** Normal control. **b** Diabetic control. **c** Diabetic + PAB (200 mg/kg). **d** Diabetic + PAB (400 mg/kg), diabetic + glibenclamide (10 mg/kg). GD, glomerular damage; TD, tubular damage; HB, hemorrhage in Bowman’s capsule; G, normal glomerulus; T, normal tubule. Number sign indicates significance from control group at *P* < 0.01 level

Histological studies of the kidney of the control group displayed normal architecture (Fig. 8a). Histology of the kidneys in diabetic rats indicated glomerular and tubular damages along with hemorrhage in the Bowman’s space (Fig. 8b). In *P. aphylla n*-butanol extract (200 and 400 mg/kg)-treated diabetic rats, minor thickness was observed in the wall of capillary loops. However, tubules and glomeruli were noticed without hemorrhage (Fig. 8 c and d). The kidney tissues of diabetic rats treated with glibenclamide showed minor fatty degenerative damages (Fig. 8e). Histopathological alterations were also noticed and recorded in score of 0–3. In diabetic rats, significantly (*P* = 0.00081) higher scores of injuries were noticed in renal tissues as compared to normal control rats. However, scores of these injuries decreased in PAB- and glibenclamide (10 mg/kg)-treated diabetic rats.

## Discussion

Diabetes is one of the non-curable diseases causing morbidity and mortality worldwide. The disease is characterized by elevated level of glucose in the blood along with dyslipidemia and oxidative stress. The long-term rise of glucose in the blood hampered the normal level and function of insulin. The pathogenesis of hyperglycemia causes complications of different organs. The disease can be managed by different ways including balanced diet, physical exercise, and lifestyle. Different medicines have showed effectiveness against diabetes mellitus including glibenclamide, metformin, and alpha-glucosidase inhibitors. However, such synthetic therapeutics have adverse effects and also not able to keep the glucose level in normal range. The local communities in the world also use different herbal products to manage and relieve the complications of type 2 diabetes on account of ease of access and with fewer side effects.

The current study makes use of the extract/subfractions of *P. aphylla* to investigate antidiabetic activity. The administration of PAB showed antihyperglycemic activity with area under a curve of 16,350 units against glibenclamide (area under a curve = 15,890 units) in glucose-loaded hyperglycemic rats. The extracts PAA (area under a curve = 17,660 units) and PAE (area under a curve = 18,010 units) also showed the hypoglycemic activity in glucose-loaded normal rats. These results showed that plant had a valuable hypoglycemic activity, and the extracts/fractions PAB, PAA, and PAE are able to inhibit the rise in postprandial level of glucose in the blood of glucose-loaded normal rats. The lowering effects on postprandial glucose with PAB and other extracts on blood glucose in glucose-loaded animals might be attributed by higher utilization of glucose for energy synthesis. Glucose-tolerance potential of *P. aphylla* implies the prevention of hyperglycemia-related complications. Numerous mechanisms of action could have been placed: enhanced peripheral utilization of glucose, insulin secretion, and insulin sensitivity. Rashid et al. [7] has reported similar antihyperglycemic effects of *Fagonia olivieri* extract in rat. HPLC-DAD analysis of PAB showed the presence of polyphenolic constituents such as rutin, catechin, caffeic acid, and myricetin. Polyphenols are a well-characterized class possessing antioxidant, antilipidemic, and antihyperglycemic properties.

Exposure of animals to STZ causes oxidative stress in β-cells of the pancreas leading to diabetes. STZ-induced diabetes is a well-recognized animal model and is frequently used to investigate the antihyperglycemic activities of drugs. In this study, different extract/fractions of *P. aphylla* were administered to glucose-loaded diabetic rats. Intake of PAB in diabetic rats significantly decreased the blood glucose with area under a curve of 62,870 units and was comparatively lower with that of other extract/subfractions. Glibenclamide also showed antihyperglycemic activity with area under a curve of 61,475 units in glucose-loaded diabetic rats. These results suggest the better glycemic control of the phytoconstituents present in PAB. Other studies also reported antihyperglycemic activity of plant extract and also of the standard drug glibenclamide [7].

On account of better antihyperglycemic activity of PAB both in normal and diabetic glucose-loaded rats, it was selected to investigate antihyperglycemic potential in a multiple-dose study for 3 weeks. Treatment of PAB at 200 mg/kg and 400 mg/kg for 3 weeks showed strong antidiabetic activity and decreased the concentration of blood glucose in rat. The area under a curve for blood glucose obtained was 3648 units with PAB (200 mg/kg) and 3370 units with PAB (400 mg/kg) treatment as compared with area under a curve of 5971 units of diabetic control rats. The higher area under a curve in diabetic control rats suggests that glucose is not efficiently used. The result obtained with PAB for area under a curve suggests that glucose is more efficiently used by these rats. Similar results were also reported in other studies where plant extracts decreased the blood glucose in diabetic animal models [7, 29]. These antihyperglycemic effects may be attributed by polyphenolics and other therapeutic constituents of PAB.

After 21 days of study period, weight of rats was recorded as a marker of general toxicity. We noted a significant decrease in body weight of diabetic control rats whereas PAB treatment to diabetic rats efficiently restored the body weight. Decrease in body weight of diabetic rats could be due to the result of decrease in ATP synthesis from carbohydrate that might be coupled with enhanced synthesis of fats and excessive breakdown of proteins. Lack of insulin in such circumstances triggers the proteolytic activity and decrease the protein content within muscle tissues. Restoration of body weight with PAB indicated the better processing of glucose as energy source and consequently decreases the muscle wastage. The increase in body weight of diabetic rat might occur as a result of increased synthesis of structural proteins, secretion of insulin in blood, and other repairing abilities of PAB constituents. Decrease in body weight of rat with STZ exposure while restoration with plant extract was also reported in previous studies [7].

In diabetic patients, morbidity and mortality are usually caused by complications associated with hyperglycemia-induced alteration in hematological parameters. In diabetic rats, level of RBC, MCV, MCHC, and MCH is significantly decreased to that of normal control rats. Peroxidation of lipids in RBCs and glycosylation of hemoglobin cause a decrease in the synthesis of hemoglobin and consequently the generation of RBCs decreases. These factors also contribute towards severe pathologies related to diabetes [30]. Amelioration of anemia in diabetic rats with PAB treatment had been recorded in this study. Such therapeutic effects could be attributed by the phytoconstituents in PAB with potential generation of RBCs from bone marrow cells in diabetic rats. These results suggested the therapeutic efficacy of PAB and can be used to restore the hematological parameters in diabetic patients. These results corresponded to previous studies where similar restoration effects on hematological parameters were recorded with plant extracts [7, 31].

In diabetic rats, decrease in count of WBCs, lymphocytes, and neutrophils was recorded after 21 days which might occur by hyperglycemic injuries that had suppressed the immune system of bone marrow. Lower platelet count in diabetic rats was also determined in this study. The normal count of platelets is very essential for proper blood clotting. In unregulated hyperglycemic diabetic patients, lower platelet count can induce ischemic stroke. Presence of such diabetic conditions for longer duration in diabetic patients can disrupt the repair of vascular injuries which may lead to death of patients as a result of internal or external bleeding. Treatment of diabetic rats with PAB restores the count of WBCs, lymphocytes, neutrophils, and platelets in blood of rat. Restoration of blood cells with this plant was in tandem with earlier reports [32]. The results suggest the presence of phytochemicals in PAB with potential therapeutic activity in restoring the count of blood cells probably by minimizing the toxic effects of hyperglycemia in diabetic patients.

In vital organs of diabetic rats, liver and kidneys, the activity of antioxidants CAT, SOD, GSH-Px, GR, and concentration of GSH decreases after 21 days of treatment. Physiological concentration of these species is required to regulate the metabolic processes of the body. TBARS in liver and kidney tissues increased in diabetic control rats. Higher concentration of glucose in diabetic control rats increases the glycosylation and ROS that causes oxidative stress and progression of diabetic complications. These conditions are usually associated with compromised antioxidant enzyme system. Higher generation of ROS decreases the secretion of insulin from pancreas. So the interventions having radical scavenging activities may be used as therapeutic agent in diabetic patients. The restoration of GR and GSH is very critical for the proper glycation and functioning of the antioxidant system. Physiological concentrations of GSH are also necessary for protection of membrane lipids against ROS. Intake of PAB by diabetic rats for 3 weeks upraised the activity of enzymatic antioxidants suggesting the antioxidant and repairing ability of PAB phytoconstituents. Among polyphenolics, the antioxidant effects of rutin had been investigated in streptozotocin-induced diabetic rat in previous studies [33]. The results suggest that use of PAB in diabetic conditions could scavenge ROS and decrease the hyperglycemia-induced complications in diabetic patients.

Enhanced oxidative stress in the liver is responsible for increased release of liver markers, AST, ALP, LDH, and ALT by virtue of damaged hepatocytes. Elevated level of these markers in diabetic rats indicated the damages of hepatocytes [34]. PAB co-administration to STZ-compelled diabetic rats exhibited repairing abilities on liver tissues and re-established the level of liver markers towards normal control rats. In other studies, restoration of liver marker enzymes is also reported with plant extracts [7, 35].

This study indicated lower level of total protein, albumin, and insulin while an increase in BUN, creatinine, and total bilirubin in serum of diabetic rats. Hyperglycemia in diabetic rats can induce renal injuries through oxidative stress. The renal injuries recorded were tubular, corpuscular, interstitial alterations, and proliferation of mesangial cells which can cause structural changes in glomerular capillaries. In diabetic rats treated with PAB, a reversion in these changes indicated the antihyperglycemic potential of phytoconstituents present in PAB. Such therapeutic effects with PAB could be achieved by repairing of β-cells and amelioration of oxidative injuries. In previous studies, an increase in insulin level in diabetic rat was also noticed [7].

It is observed that circulation of free fatty acids is enhanced in diabetic patients. The free fatty acids have deleterious effects on the endothelial function and are usually achieved by different mechanisms among which ROS production is the key mechanism. Under normal circumstances, triglycerides are hydrolyzed by lipoprotein lipase while insufficiency of insulin causes hypertriglyceridemia and hypercholesterolemia. In this study, dyslipidemia conditions such as hyperlipidemia, higher level of triglycerides, total cholesterol, and LDL while lower level of HDL, were recorded in diabetic rats. Dyslipidemia in diabetic rats was also recorded in previous studies [36]. Insulin resistance in diabetic patients can trigger the synthesis of glucose and triglycerides from liver tissues and is associated with enhanced generation of superoxide radicals [37]. Further, lower level of insulin triggers higher lipolytic activity and consequently enhanced the synthesis of cholesterol and LDL particles which leads to micro and macro cardiovascular complications. In this study, PAB treatment to diabetic rats significantly decreased the triglycerides and cholesterol levels. It has been well characterized that development of atherosclerosis can be prevented by increasing the level of HDL in diabetic conditions. Similar hypolipidemic activity was also reported in previous study [7]. The results of this study support the hypothesis that PAB may directly impact an improvement on insulin level and thereby decrease diabetic complications by restoring the metabolism of lipid profile towards the normal level.

The metabolic alterations and oxidative stress induced by hyperglycemia can cause inflammation in diabetic conditions. Level of TNF-α, IL-6, and TGF-β1 significantly increased in serum of diabetic control rats. Higher level of TGF-β1 can be induced by higher concentration of ROS and consequently induce glomerular injuries of the kidneys of diabetic individuals. Resistance against insulin is a common phenomenon in diabetes type 2 patients that is usually associated with aberrant signaling of insulin [38]. Such alterations in metabolism elicit the generation of superoxide radicals in mitochondria leading to hepatic injuries. Further, inflamed hepatocytes along with release of other metabolites mediated the synthesis of connective growth factor and collagen by activation of stellate cells and thereby causing fibrosis [39]. Liver histopathology of diabetic rats in this study showed severe abnormalities including fatty deposition, vacuolization, and pyknotic nuclei. Hepatic injuries in diabetic animals had also been reported in previous studies [7]. In this study treatment of diabetic rats with PAB ameliorated the oxidative stress, restored the pro-inflammatory cytokines, and also decreased the hepatic injuries. Administration of rutin an important polyphenol to diabetic rats maintained the level of inflammatory cytokines and ameliorated the histological alterations in renal tissues of rat [33]. The study suggests the potential therapeutic ability of PAB to improve glycemic status and prevent the diabetic-associated complications in diabetic patients.

## Conclusion

Results of our study indicate that among the extract/fractions the butanol fraction (PAB) of methanol extract of *P. aphylla* strongly exerted the antihyperglycemic activity. The administration of PAB to STZ-prompted rat maintains the level of various metabolites of the liver, pro-inflammatory cytokines, and antioxidant enzymes and diminished the cellular injuries of the liver and kidneys through scavenging of free radicals.

## Data Availability

The data may be available on request.

## Abbreviations

PAM: *P. aphylla* methanol extract
PAH: *N*-hexane fraction of PAM
PAC: Chloroform fraction of PAM
PAE: Ethyl acetate fraction of PAM
PAB: *N*-butanol fraction of PAM
PAA: Residual soluble aqueous fraction of PAM
AST: Aspartate transaminase
ALT: Alanine transaminase
ALP: Alkaline phosphatase
LDH: Lactate dehydrogenase
MCV: Mean corpuscular volume
MCHC: Mean corpuscular hemoglobin concentration
MCH: Mean corpuscular hemoglobin test
TLC: Total leukocyte count
PLT: Platelet count
TNF-α: Tumor necrosis factor-alpha
IL-6: Interleukin-6
TGF-β1: Transforming growth factor-beta 1
GR: Glutathione reductase
GPx: Glutathione peroxidase
SOD: Superoxide dismutase
TBARS: Thiobarbituric acid reactive substances
BUN: Blood urea nitrogen
LDL: Low-density lipoprotein
HDL: High-density lipoprotein
